# Cell Replacement Therapy for Retinal and Optic Nerve Diseases: Cell Sources, Clinical Trials and Challenges

**DOI:** 10.3390/pharmaceutics13060865

**Published:** 2021-06-11

**Authors:** Rosa M. Coco-Martin, Salvador Pastor-Idoate, Jose Carlos Pastor

**Affiliations:** 1 Instituto de Oftalmobiologia Aplicada (IOBA), Medical School, Universidad de Valladolid, 47011 Valladolid, Spain; pastoridoate.salvador@gmail.com (S.P.-I.); pastor@ioba.med.uva.es (J.C.P.); 2 National Institute of Health Carlos III (ISCIII), (RETICS) Cooperative Health Network for Research in Ophthalmology (Oftared), 28040 Madrid, Spain; 3 Department of Ophthalmology, Hospital Clinico Universitario of Valladolid, 47003 Valladolid, Spain; 4 Centro en Red de Medicina Regenerativa y Terapia Celular de Castilla y León, Fundacion del Instituto de Estudios de Ciencias de la Salud de Castilla y León (ICSCYL), 42002 Soria, Spain

**Keywords:** stem cells, retinal diseases, optic nerve diseases, cell replacement, cell sources

## Abstract

The aim of this review was to provide an update on the potential of cell therapies to restore or replace damaged and/or lost cells in retinal degenerative and optic nerve diseases, describing the available cell sources and the challenges involved in such treatments when these techniques are applied in real clinical practice. Sources include human fetal retinal stem cells, allogenic cadaveric human cells, adult hippocampal neural stem cells, human CNS stem cells, ciliary pigmented epithelial cells, limbal stem cells, retinal progenitor cells (RPCs), human pluripotent stem cells (PSCs) (including both human embryonic stem cells (ESCs) and human induced pluripotent stem cells (iPSCs)) and mesenchymal stem cells (MSCs). Of these, RPCs, PSCs and MSCs have already entered early-stage clinical trials since they can all differentiate into RPE, photoreceptors or ganglion cells, and have demonstrated safety, while showing some indicators of efficacy. Stem/progenitor cell therapies for retinal diseases still have some drawbacks, such as the inhibition of proliferation and/or differentiation in vitro (with the exception of RPE) and the limited long-term survival and functioning of grafts in vivo. Some other issues remain to be solved concerning the clinical translation of cell-based therapy, including (1) the ability to enrich for specific retinal subtypes; (2) cell survival; (3) cell delivery, which may need to incorporate a scaffold to induce correct cell polarization, which increases the size of the retinotomy in surgery and, therefore, the chance of severe complications; (4) the need to induce a localized retinal detachment to perform the subretinal placement of the transplanted cell; (5) the evaluation of the risk of tumor formation caused by the undifferentiated stem cells and prolific progenitor cells. Despite these challenges, stem/progenitor cells represent the most promising strategy for retinal and optic nerve disease treatment in the near future, and therapeutics assisted by gene techniques, neuroprotective compounds and artificial devices can be applied to fulfil clinical needs.

## 1. Introduction

Retinal degenerative diseases (RDs) have been largely characterized and are considered leading causes of blindness worldwide. They include age-related macular degeneration (AMD) and inherited retinal dystrophies (IRDs) such as retinitis pigmentosa (RP) and Stargardt’s disease [1,2]. There is also some retinal degeneration associated with ischemic disorders such as diabetic retinopathy (DR) and retinal vascular occlusion (RVO), which are also relevant to this review [3,4]. All these disorders share some common pathophysiology pathways that lead to the early loss or dysfunction of photoreceptors and/or neural apoptosis. As part of the central nervous system (CNS), the retina has very low regenerative capability, which can result in untreatable blindness [1,2,3,4]. The available therapies for some RDs can protect retinal neurons, rescue or slow disease progression or relieve symptoms, but currently there are hardly any treatments to restore vision, because at present, lost cells cannot be replaced. Stem cell-based therapy is an exciting, rapidly advancing area of translational research that has already entered the clinic. Some of the advantages of the eye as a target organ for cell-based therapy—mainly for the retina—are the following. Its anatomy and physiology are very well known; surgical techniques to access the retina are well established and are reasonably safe (in fact, they are routine clinical procedures everywhere); the subretinal space is a relatively immune-privileged site; the number of cells needed to restore vision may be relatively small; retinal imaging in the living human eye is available with high resolution noninvasive techniques; fellow eye can be used as a control; finally, electrodiagnostic and psychophysical testing to assess functional recovery are also available and well characterized [5].

Rescue strategies seeking a trophic effect from stem/progenitor cell treatment have been investigated, but their efficacy and efficiency are generally restricted by the low rate of proliferation and/or differentiation of cells in vitro and by poor cellular survival, migration, integration and function in vivo, excluding RPE-based therapy for RD. Nevertheless, cell therapy assisted by gene techniques, neuroprotective compounds and artificial devices can be used in these diseases to fulfil clinical needs.

Regarding cell therapy in optic nerve diseases (ONDs), the situation has progressed little in the last five years, compared to a review carried out by our group [6]. We believe there are several reasons. On the one hand, this is due to the heterogenicity of the pathologies that researchers have tried to treat with cell therapy, which in many cases affect some other parts of the CNS too, and it is also due to the obvious difficulties of access in delivering any treatment to some anatomic sections of the optic nerve. It should not be forgotten that the optic nerve (ON) is “born” in the retinal ganglion cells (RGCs) and extends to the lateral geniculate nucleus [7]. The category of so-called optic neuropathies includes a broad spectrum of diseases with various causes, including ischemia, inflammation, toxicity, nutritional deficiencies, glaucoma, trauma, congenital problems and hereditary diseases, in most cases as part of wider neurodegenerative processes [8]. The ON is basically composed of RGC axons, and like other adult neurons of the CNS, they do not have the ability to regenerate after injury. Many factors limit the regeneration of RGC axons. Some are derived from the inhibitory environment created after RGCs suffer axonal damage. Furthermore, oligodendrocytes secrete inhibitory proteins and other molecules which impede axon regrowth, unlike myelinating Schwann cells that promote axon regeneration in the peripheral nervous system, but these cells are not present in the ON. Astrocytes also release inhibitory molecules and proliferate, creating glial scars acting as physical barriers to axonal regeneration [9,10,11]. Moreover, many genes which are necessary for cellular proliferation and axon growth, although active in embryonic cells, are deeply suppressed in mature ones [9,10]. Finally, axonal injury also interrupts the transport of neurotrophic factors, resulting in an increase in proapoptotic proteins in RGCs [12,13,14].

Reviews on the use of intravitreal cell therapy to confer neuroprotection through their paracrine properties have been published by our group [6,15]. Several phase I and II clinical trials (CTs) have demonstrated the safety of many types of stem cells, and this fact has prompted researchers to continue to progress in the exploration of the efficacy of this approach [15,16]. However, there are still many unknowns to be solved, such as the best source of cells, the best route of administration, the possible means of inducing the regeneration of lost cells and above all how to maintain the possible beneficial effects in the long term [16]. These unknowns have to be resolved before any of the treatments become a regular part of clinical therapies. In this review, we provide an update on the potential of cell therapies to restore or replace damaged and/or lost cells in RD and OND, reviewing the available data on published CTs and describing the available cell sources and the challenges involved in applying such treatments in real clinical practice.

## 2. Search Strategy and Selection Criteria

This review cited CTs performed on cell therapy published in the PubMed, Web of Science, Scopus and ClinicalTrials.gov (accessed on 2 May 2021) electronic databases in the most recent years up to December 2020. Potentially relevant papers were obtained using the following search terms in combination as Medical Subject Headings and text words: human, stem cell, cell therapy, clinical trials, intraocular injection, intravitreal injection, subretinal injection, retina, retinal diseases, optic nerve and optic nerve diseases. Only English papers or those with an English abstract were preselected. The reference lists of the selected publications were also scanned to identify additional relevant papers and the MEDLINE option “Related Articles” was also used.

## 3. Cell Sources

Embryonic cells within the first couple of cell divisions after fertilization are the only cells that are totipotent and they can form all the cell types in a body, plus the extraembryonic or placental cells. Pluripotent cells can give rise to all of the cell types that make up the body; embryonic stem cells are considered pluripotent. Finally, multipotent cells can develop into more than one cell type but are more limited than pluripotent cells; adult stem cells and cord blood stem cells are considered multipotent. Retinal progenitor stem cells (RPCs) are also multipotent cells that can give rise to all the six neurons of the retina and the Müller glial cells.

The main sources of stem cells for transplantation are summarized in Table 1. They include human fetal retinal stem cells, allogenic cadaveric human cells, adult hippocampal neural stem cells, human CNS stem cells, ciliary pigmented epithelial cells, limbal stem cells, RPCs, human pluripotent stem cells (hPSCs) including both human embryonic stem cells (ESCs) and human induced pluripotent stem cells (iPSCs), and mesenchymal stem cells (MSCs) [17,18]. Finally, cells extracted from the adult human RPE, obtained from eye banks and activated in vitro into a stem cell state (RPESCs), are a potential source of such cells [19]. Of these, RPCs, PSCs and MSCs have demonstrated their ability to assume some of the functions of native tissue and have all been used in an increasing number of CTs, since they all can differentiate into RPE, photoreceptors or ganglion cells [20].

**Figure 1:**
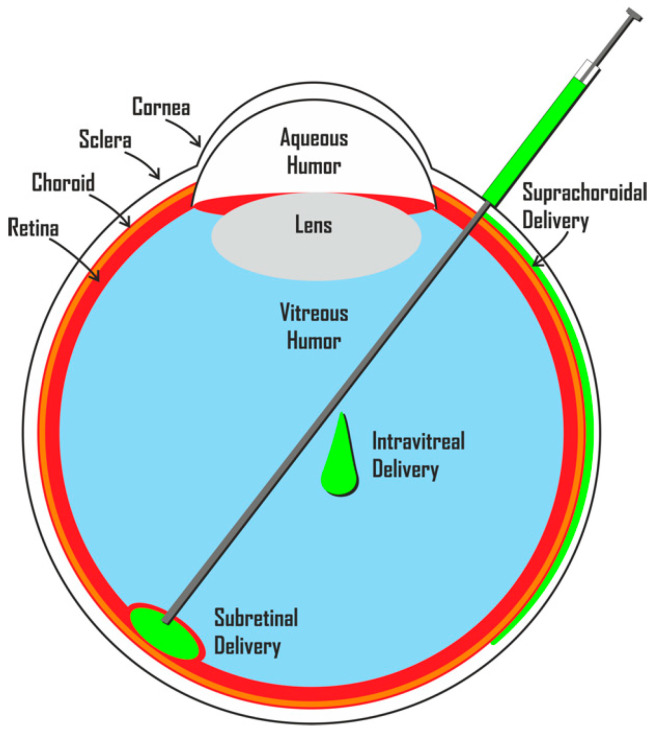
Cell delivery can be either suprachoroidal, intravitreal or subretinal.

Routes of delivery (suprachoroidal, intravitreal and subretinal) are presented in Figure 1.

RPCs are obtained from the fetal and postnatal retina. Their main advantages are their simple accessibility, safety and effectiveness, the fact that they are widely studied, they avoid ethical issues and have low risk of immune rejection and tumorigenesis. Their main disadvantages are the shortage of sufficient donor cells due to their limited proliferative capacity and their restricted ability to differentiate into specific types of cells [21].

MSCs may originate from amniotic fluid or the umbilical cord, although they are mainly obtained from two developmentally mature organs: bone marrow mesenchymal stem cells (BMMSCs) and adipose mesenchymal stem cells (ADMSCs). The latter are much more abundant and easier to harvest from alive donors, with less invasive procedures. Moreover, they expand faster and demonstrate a higher immunomodulatory capacity than BMMSCs. MSCs have been shown to have anti-inflammatory, immunosuppressive, angiogenic and antiapoptotic or neuroprotective effects [22,23]. Furthermore, they are multipotent; thus, they have some ability to differentiate into damaged cells, although this is somewhat limited. They have a low rate of cell migration and differentiation, though have been reported to differentiate into photoreceptors and RPE cells. Nevertheless, it remains unclear if the newly observed cells may represent the fusion of MSCs with pre-existing photoreceptors [21,24].

Human ESCs come from developing embryos. Their main advantage is their ability to differentiate into photoreceptors under certain circumstances, creating an unlimited source of cells for RD treatment, whereas their main disadvantages are their limited proliferation and multidifferentiation into various cell types, thus presenting difficulties in obtaining the specific targeted cell type; their potential for tumor formation; the requirement of lifelong immunosuppressive therapies that increase risks and economic burdens; finally, since ESCs are isolated from fetal tissues, they raise ethical concerns [25].

The need to provide large numbers of replacement cells has tipped the process toward the use of iPSCs. Various groups have developed protocols to induce and reprogram these cells since their introduction in 2006, and the next challenge will be to establish guidelines to determine their quality [26]. They are obtained from terminally differentiated tissues, which ameliorate the ethical issues of ESCs. They also have a low risk of immune rejection through autologous transplantation, with the disadvantage of a low differentiation efficiency despite their similarity to ESCs, as well as biosafety concerns (e.g., the high risk of gene mutations) [21]. Furthermore, iPSCs have been critical in advancing our understanding of the underlying mechanisms (ontogenesis and pathology) of numerous retinal and optic nerve disorders such as AMD, RP and glaucoma. Cell models have been developed using iPSCs, and these are also important in the study of retinal disease, as well as in developing drug screening and gene therapy approaches. Finally, a new iPSC-based therapy for RD in humans was first reported by a Japanese group in 2017 [26].

On the other hand, stratified neural retina and RPE in a single complex could also be a potential tool in the development of a dual RPE/photoreceptor graft that could be used in individuals with end-stage RD. Recent studies have shown the formation of entire optic cups from ESCs in minimal media conditions [27]. Given the difficulty of the derivation of photoreceptors, especially for producing mature outer segments in 2D cultures [27], approaches using 3D retinal organoid cultures have been attempted. Considerable progress has been shown in the growth of self-organized 3D optic cups from human ESCs, which showed the formation of photoreceptors with reasonable inner segments and connecting cilia [27]. Other methods used for 3D organoid formation have been reported, mechanically picking them up from 2D cultures during differentiation and further optimizing them using a two-step culture system for human iPSCs [28]. Finally, there are also protocols for generating 3D optic vesicle-like structures from human iPSCs showing axon growth [29].

In the case of retinal vasculopathies, direct tissue replacement might be more challenging, as different cells are involved in their pathogenesis, such as the vascular endothelium cells, vascular pericytes, vascular smooth muscle cells, inner retinal neurons, photoreceptors and the retinal glia and microglia cells. Most researchers used MSCs and RPCs, which are considered to have some (although limited) ability to differentiate into the various cells damaged in the context of retinal vascular disease [30,31]. Thus, circulating vascular precursor cells CD34+ and endothelial progenitor cells (EPCs) have been used for tissue regeneration and angiogenesis following ischemia due to the fact that they may play a role in functional collateralization and secrete neurotrophic cytokines and proangiogenic factors [30,32]. CD34+ may differentiate into endothelial cells and because of this they are being explored in CTs as a potential therapy for various ischemic disorders, including ischemic cardiomyopathy, peripheral ischemia, cerebrovascular accidents, DR, ischemic retinal vein occlusion and ischemic optic neuropathy [33,34,35]. Another potential cell source is a subpopulation of EPCs named outgrowth endothelial cells (OECs), which have significant proliferative potential, but which need to be explored further as a therapy for ischemic retinopathies [36]. In addition, within the stromal vascular fraction of ADSCs there is a distinct population of cells that are thought to represent resident pericytes or their precursors. When these cells were administered intravitreally and intravenously into animal models of oxygen-induced retinopathy (OIR) and DR, the perivascular integration of these cells was observed producing the rescue of damaged retinal capillaries [37]. One more approach would be to use subretinal transplantation of iPSCs (without c-Myc to minimize teratogenicity), because in rat eyes they were able to rescue the ischemic damaged retina through trophic paracrine effects. Moreover, researchers differentiated ESCs or iPSCs into endothelial precursor cells (particularly, endothelial colony unit-forming cells), and these showed some efficacy in treating an animal model of OIR [38]. Nevertheless, in a murine model of ischemia-reperfusion injury, vascular progenitor cells derived from ESCs and iPSCs from cord blood showed engraftment, homing and repair capabilities, whereas those derived from fibroblasts did not [39]. Lastly, extracellular stem cell-derived exosomes (MSC-Exos) may have a positive role in anatomical and functional restoration of the retina in retinal ischemia and DR by modulating angiogenesis and inflammation pathways, through immunomodulation or even through tissue regeneration [40,41].

## 4. Direct Cell Replacement Therapy for Retinal and Optic Nerve Diseases

### 4.1. RPE Replacement

Human RPE cells were first isolated and characterized over 30 years ago, and since that time, cell replacement has been tested as a potential treatment for RD. The second attempt at these cells’ replacement occurred during the last decade, when RPE derivation from ESCs and iPSCs was established in many laboratories. The RPE cell layer does not require synaptic connections, unlike other cell types in the retina, but its ability to perform its essential functions depends on the RPE being a confluent monolayer with tight junctions and maintaining polarity for ion transport with a healthy Bruch’s membrane [42,43]. Nevertheless, subretinal injection of healthy RPE cells allows them to maintain or improve the health of the outer nuclear, outer plexiform and photoreceptor inner/out segment layers [44]. An advantage of this is, as was mentioned before, that the subretinal space is a unique target for cell-based therapy because it is an immune-privileged environment in normal conditions. Therefore, direct cell replacement is being explored as a potential therapy for macular atrophy, using stem cells injected into the submacular space, since RPE dysfunction and death in the macula is the main devastating feature of AMD and Stargardt’s disease. This kind of cell therapy has also been launched for RP patients with monogenic mutations affecting RPE65, LRAT and MERTK, genes involved in visual signaling process dysfunction, specifically at the RPE level [45].

Clinical trials on RPE replacement are numerous and a great number of them have succeded (Table 2). A cell product named CNTO2476, consisting of a suspension of human umbilical multipotent stem cells retrieved from donor umbilical cords (hUTSCs), has also been subretinally injected in patients with geographic atrophy due to AMD in a clinical trial (NCT01226628). The trial has been completed but its results have not been posted. Moreover, there are two open labeled CTs evaluating the safety and efficacy of autologous BMMNCs in the subretinal space of patients with RP (NCT01914913 and NCT02280135). These studies have not yet provided definitive data, but the preclinical results are promising in RPE diseases, in which the morphology of photoreceptors has seemed to improve. Two more CTs using BMMSC intravitreally are ongoing for RP patients (NCT01560715 and NCT01531348). However, another CT has raised concerns about the safety of BMMSCs, as one out of three patients with advanced RP developed severe fibrous tissue proliferation at the injection site, in the vitreous cavity and in the retrolental space, which led to tractional retinal detachment [46]. One more ongoing CT in Saudi Arabia (NCT02016508) is investigating the safety and efficacy of the unilateral intravitreal injection of autologous BMMSC in subjects with geographic atrophy secondary to AMD [47].

Concerning hESCs, two cell products are being tested in CTs for macular diseases, one from Pfizer (NCT01691261) and the other from the Astellas Institute for Regenerative Medicine (formerly Ocata/Advanced Cell Technology). The latter is currently completing a phase I/IIa CT designed to test the tolerability of transplanted RPE cells derived from hESCs for the treatment of patients with Stargardt’s disease (NCT01345006) and advanced dry AMD (NCT01344993) without a control group and using systemic immunosuppression. Indications of effectiveness have been shown, as 10 out of 18 patients improved their vision. There was no evidence of proliferation, rejection or serious systemic adverse events, but one patient had staphylococcus endophthalmitis, and cataract progression. Localized RPE damage and intraocular inflammation were reported [48,49]. In addition, transplantation of hESM-derived RPE cells in the subretinal space with systemic immunosuppressive therapy for 13 weeks has been tested in 12 patients with Stargardt’s disease (NCT01469832) using the same biological. In that trial, focal areas of subretinal hyperpigmentation were observed in all participants in a dose-dependent manner and no evidence of uncontrolled proliferation or inflammatory responses were found. Borderline improvements in best-corrected visual acuity (BCVA) in four participants either were unstained or showed a similar improvement in the untreated contralateral eye. Quality of life questionnaires and microperimetry demonstrated no evidence of a benefit at 12 months, and in one case, localized retinal thinning and reduced sensitivity in the area of hyperpigmentation suggested potential harm [50]. Lineage cell therapeutics has developed a similar study in patients with the advanced atrophic-form of AMD (still recruiting) that has the objective of evaluating the safety and tolerability of a cell product named OpRegen^®^ (hESC-RPE cells) transplanted subretinally via the suprachoroidal approach through a microinjection using the Orbit Subretinal Delivery System (Orbit SDS) developed by Gyroscope Therapeutics (formerly Orbit Biomedical, Ltd.), which avoids the need to create a retinal hole and aims to provide precise and consistent dosing. The study will also assess the ability of transplanted OpRegen^®^ cells to engraft, survive and moderate disease progression (NCT02286089) and results will be presented later this year. Furthermore, there are several recruiting phase I/II clinical trials using hEMS in AMD and/or Stargardt’s disease in China (NCT02749734, NCT03046407 and NCT02755428), the United States (NCT01344993, NCT02463344, NCT03167203, NCT01345006 and NCT02445612), France (NCT02941991 and NCT01469832) and Korea (NCT01625559 and NCT01674829) aiming to verify the overall safety and feasibility of hESC-RPE cell-based therapies, providing some promising early visual results, in which any major complications could be primarily attributed to the use of immunosuppressants during allogenic transplantation [45,51,52]. In addition, for RP a trial using hESC-derived-RPE cells is recruiting 10 patients to test the safety and efficacy of its subretinal transplantation technique (NCT03944239). Finally, a phase I/II, open-label, prospective CT tried to determine the safety and tolerability of the subretinal transplantation of hESC-derived RPE cells (MA09-hRPE) in patients with patchy atrophy secondary to myopic macular degeneration (NCT02122159), but the study was withdrawn in 2016 and no results have been posted.

RPE cells obtained from human autologous somatic cells (hiPSCs) were used in a phase I CT in 2013 in Japan (UMIM000011929) in which the first patient improved without adverse effects, but the study was put on hold because oncogenic genetic mutations were found in the second patient, probably due to the documented genomic instability of iPSCs. The CT since resumed, using HLA-matched allogenic iPSCS-derived RPE cells in suspension compared with autologous iPSCs, the first being safer and more likely to succeed economically. The transplanted sheet remained intact and BCVA was stable one year after surgery, although cystoid macular edema was present [53]. More recently, another two ongoing CTs have commenced in England and the USA (NCT02464956) to test the efficiency of creating iPSC-derived RPE cells from the patient’s own skin or blood. This trial started in 2015 in 10 patients with AMD and is not yet recruiting [54]. At the same time, many scientists are studying the safety concerns surrounding the iPSCs obtained through the reprogramming process.

Recently, a combination of gene and cell therapy has been implemented using the CRISPR/Cas9 system applied to the production of iPSCs with selective HLA gene disruption [55]. One example is a recent CT for a specific type of RP with mutations in disease-causing genes that affect RPE function (NCT03963154), aiming to restore RPE function and protect photoreceptors from degeneration at a relatively early stage.

However, the delivery strategy of a cell suspension might not be sufficient, and more complex reconstructed tissue formulations are probably required, both to improve functionality and to target pathological conditions with altered Bruch’s membrane-like AMD. For clinical applications, Kamao et al. developed a protocol for an RPE monolayer sheet obtained from hiPSC-RPE cells without using any synthetic scaffold, but rather self-producing their basement membrane consisting of collagen IV and laminin. This was shown to be functional in vivo when used in neovascular AMD after the removal of the choroidal neovascularization, with no sign of rejection and no patients needing additional anti-VEGF injections. The major problem was the cost and extensive preparation time needed for each individual patient (more than 10 months) [56,57]. Likewise, more complex reconstructed tissue formulations have been proposed in order to improve functionality and replace the damaged Bruch’s membrane in AMD using 3D bioengineered tissues amenable for regenerative medicine, developing RPE sheets or substrates to make the technical transfer more tolerable to the cells during surgery and to increase survival compared to cells in suspension, as well as increasing the chance of the cells forming an organized orientation tissue reminiscent of the endogenous cellular structure [58]. An example of this new strategy is a recruiting phase I/II CT (NCT02903576) that is trying to determine whether the surgical implantation of an hESC-derived RPE cell monolayer seeded onto a polymeric substrate versus hESC-derived RPE cell injections alone into the subretinal space are safe procedures. This has been planned in patients with dry AMD, disciform scarring due to wet AMD and Stargardt’s disease. Thus, Kashani et al. have designed an implant using a scaffold, termed the California Project to Cure Blindness–Retinal Pigment Epithelium 1 (CPCB-RPE1), which consists of a polarized monolayer of hESC-derived RPE cells on an ultrathin synthetic parylene (plastic) substrate designed to mimic Bruch’s membrane. This group has published data on a cohort of 16 patients with advanced dry AMD (NCT02590692), which demonstrate the technique’s safety and suggest that it may improve visual function, since none of the implanted eyes showed the progression of vision loss, one eye improved by 17 letters and two eyes demonstrated improved fixation [59]. The group led by da Cruz et al. is investigating a similar RPE patch in severe exudative AMD as a part of The London Project to Cure Blindness (NCT03102138). Finally, a phase I CT used an engineered RPE patch comprising a fully differentiated hESC-derived RPE monolayer on a coated, synthetic basement membrane, delivered using a purpose-designed microsurgical tool into the subretinal space of one eye in two patients with severe exudative AMD. Only local immunosuppression was used long-term. The authors reported the successful delivery and survival of the RPE patch by means of biomicroscopy and optical coherence tomography (OCT), and a BCVA gain of 29 and 21 letters in the two patients, respectively, over 12 months. They also presented the preclinical surgical, cell safety and tumorigenicity studies, leading to the trial’s approval [60].

### 4.2. Photoreceptor Replacement

To treat IRD, and mainly RP, it is necessary to replace dysfunctional or dead rods and cones, creating a therapeutic “slot” for cell therapies between gene therapy (for early stages of the disease) and retinal microchips (for advanced stages of the disease). One of the difficulties in treating advanced stages is that most of these conditions affect the entire retina. Replacing photoreceptors has been tried when these are the major cell type involved in retinal degeneration. In this case, the introduced precursors would have to form a polarized outer nuclear layer with the formation of light-sensitive outer segments, and then would have to reconnect synaptically with downstream retinal neurons in order to send information down the visual pathway.

To do this, various forms of transplant have been applied, including a full-thickness retina; photoreceptor sheets (sliced using a laser or vibratome); dissociated cells, including photoreceptors or the retinal progenitor cells (RPCs) that are able to produce them; hPSC-derived cells [61]. Patients may also benefit from a combined RPE and photoreceptor transplant, but whether this would be a stepwise approach with RPE replacement followed by photoreceptor transplantation or whether the two could be transplanted together needs to be explored, as Zhu et al. showed that the survival of photoreceptor progenitor cells was increased when they were cocultured with hESC-derived RPE [62].

The first publications on this issue indicated the potential benefit of transplanting fetal retinal cells or tissue in patients with retinal degeneration and its safety, but these grafts were limited due to ethical concerns and reduced availability [63]. Furthermore, the surgical techniques used to perform the subretinal transplantation of full-thickness retina or photoreceptor sheets are difficult to perform, and cell integration and synaptic reconnection are also challenging [64]. However, with the advent of well-established protocols to differentiate substantial quantities of retinal cells from hESCs and iPSCs, regenerative retinal therapies have become a practical goal in clinical practice [65].

RPCs have been demonstrated to become mature and express photoreceptor markers when injected into the subretinal space, and they are also able to integrate into the host inner retina and rescue degenerated photoreceptors [66]. RPCs and hESC-derived photoreceptor precursor cells have been shown to integrate into the host retina and improve light sensitivity, although the effect was reversed in months [66,67]. Another source could be hiPSC-derived photoreceptor precursors—results have demonstrated that adult fibroblast-derived iPSCs can differentiate into retinal precursors to be used for the transplantation and treatment of retinal degeneration diseases [68,69,70]. The main question is whether transplanted photoreceptors actually integrate. They may instead fuse with existing photoreceptors, since in recent years a phenomenon known as “material transfer” has been proposed, whereby biomaterial such as proteins and/or mRNA is transferred from donor to host photoreceptors, thereby restoring some visual function by rescuing remaining photoreceptor cells [71,72]. An important point is that more progress in preclinical studies is needed in order to better understand and optimize cell integration in order to plan future CTs.

CTs on photoreceptor replacement are summarized in Table 3. One of them is implementing the use of subretinally transplanted hRPCs derived from the fetal retina. This is a phase I/IIa, open-label, prospective study aiming to test their safety and tolerability in patients with advanced RP and has been sponsored by ReNeuron (NCT02464436).

Mesenchymal stem cells have a reduced ability of cell differentiation when compared to embryonic stem cells although they may differ in some cells such as retinal pigmented epithelium cells and retinal glial cells. However, these cells secrete large amounts of trophic factors that could theoretically increase the longevity of retinal cells in distress and also to produce a recovery of function. With this goal, Siqueira et al. at the University of Sao Paulo have primarily investigated the use of autologous BMMSCs intravitreally injected to treat patients with advanced degenerative retinopathies (one RP patient and two affected by cone-rod dystrophy) in a phase I CT (NCT01068561), without detecting serious adverse events. In a phase II study, they started to confirm the efficacy of this technique (NCT1560715) in 20 RP patients, showing a transitory improvement of vision that lasted no longer than one year [73,74]. The same group has investigated the safety and effectiveness of their cell product in AMD and Stargardt’s patients (NCT01518127). Nevertheless, this is not real photoreceptor replacement but rescue.

Another approach is to use iPSCs, but in the particular case of IRD, patient-derived iPSCs carry pathogenic gene mutations that may affect the survival and function of autologous transplanted cells. Thus, the cell replacement strategy can utilize patient-specific photoreceptor precursor cells that have been genetically corrected through conventional gene therapy using viral vectors or via gene editing, using both the clustered regularly interspaced short palindromic repeats-associated protein 9 (CRISPR/Cas9) system or the transcription activator-like effector nucleases (TALEN) system [75,76]. However, the phenotypic correction of iPSCs is not efficient enough (because of transgene silencing), so it may be more advantageous to correct somatic cells “ex vivo” before reprogramming. The advantage of this approach is that it could be used to treat IRD, regardless of the clinical stage or prevalence of the disease, and of the size of the causative gene [77]. Further work is required to ensure safety regarding off-target mutations due to gene editing and mutagenesis that may occur during the derivation and differentiation of iPSCs, although despite these challenges, gene editing technology has made rapid advances and is a valuable tool in understanding and treating RD [78]. CRISPR/Cas9 can be also used to turn genes on, instead of snipping them via epigenetics, by modulating histone marks, rather than editing DNA sequences, thus obtaining improvements or the amelioration of symptoms. Nevertheless, some challenges remain before this can be implemented in the clinic [79]. Additionally, a drug-tunable gene therapy, which led to the expression of a neurotrophic factor-destabilization domain fusion protein, preserved cone vision in preclinical studies, suggesting its potential use against broad-spectrum RD and its possible use as an adjunct therapy along with stem-cell therapy [80]. In this respect, Cereso et al. used AAV2/5 as a carrying vector to effectively transduce iPSC-derived RPE cells from a choroideremia patient, thus illustrating the potential of patient iPSC-derived RPE cells to provide a proof-of-concept model for gene replacement when there is no appropriate animal model [81]. Furthermore, Burnight et al. transduced patient-specific, iPSC-derived, photoreceptor precursor cells with lentiviral vectors carrying full-length CEP290 in order to correct a causing mutation of Leber’s congenital amaurosis, which affects the cilia formation of the photoreceptors. Their results showed the expression of full-length transcripts and functional rescue of the ciliogenesis defect in patient cells [82]. Bassuk et al. used the CRISPR/Cas9 system to precisely repair an RPGR point mutation that causes X-linked RP (XLRP) [83]. Lastly, another CT using iPSCs to develop cell models of different retinal dystrophies is also recruiting (NCT03853252) to evaluate the efficiency of gene therapy approaches.

An additional approach to cell-based therapy is to introduce optical sensors into grafted photoreceptor cells to make them function stably and independently of the RPE [84].

Finally, mesenchymal stem cell-derived exosomes are also being tested in a clinical trial (NCT03437759) because they seem to the promote healing of large and refractory macular holes.

### 4.3. Ganglion Cell Replacement and Cell Therapy for Optic Nerve Diseases (ONDs)

Among the studies registered in ClinicalTrials.gov (accessed on 2 May 2021), only 18 are related to OND, and six have not shown results for a long time, so it is presumable that they have failed or have been interrupted (Table 4). The rest are phase I or II CTs related to a few diseases (Table 5).

Three of them are focused on optic nerve atrophy, which is the end result of many pathologies with different pathogeneses. Four included patients with optic neuromyelitis (Devic’s disease), an autoimmune disorder predominantly characterized by severe optic neuritis and transverse myelitis. For many years this disease was considered a variant of multiple sclerosis, but the discovery that most patients have autoantibodies against aquaporin-4 (AQP4) or NMO-IgG changed the understanding of the disease [85].

In one of the CTs, dominant optic atrophies and Leber’s hereditary optic neuropathy (LHON) are included, although many other ocular pathologies are also included. This trial will be discussed below.

Three of the CTs are focused on glaucoma. This disease has traditionally been viewed as a primary OND, in which the optic nerve is damaged as a result of high intraocular pressure (IOP). Glaucomatous optic neuropathy is characterized by significant death of RGCs. According to global surveys, the second leading cause of blindness after cataracts is glaucoma. However, there is a substantial group of people (up to 20%) with typical glaucomatous disc changes, progressive visual field defects and open anterior chamber angles associated with intraocular pressure (IOP) constantly below 21 mmHg, a condition known as normal tension glaucoma [86]. Currently the main goal of glaucoma treatment is IOP reduction. The Early Manifest Glaucoma Trial showed that glaucoma progression was decreased by 10% with the reduction of each mmHg of IOP but according to the Collaborative Normal Tension Glaucoma Study Group, an IOP reduction of 30% is required to slow the progression of normal tension glaucoma—a goal that is difficult to achieve with the currently available glaucoma treatments [86]. Therefore, treatment should be ideally targeted at neuroprotection to improve the RGCs or optic nerve head function by means of drugs such as calcium channel blockers or by means of cell therapy. In this case, cell transplantation is still at an early stage of preclinical study, compared with RPE or photoreceptor transplantation. Finally, a CT sponsored by our group (NCT03173638) is focused on the acute phases of acute nonarteritic anterior ischemic optic neuropathy [6].

Regarding the types of cells, six CTs use BMMSCs. Three of these—all of them directed at optic neuromyelitis—use a combination of a high dose of immunosuppressive therapy, followed by autologous hematopoietic cell transplantation.

Two CTs use encapsulated cell technology. They use ARPE19, a retinal pigmented human cell line, genetically modified to produce ciliary neurotrophic factor (CNTF). Cells are encapsulated in a semipermeable polymer capsule which is introduced into the vitreous cavity. The idea seems very attractive and in theory it would open up many possibilities. It was primarily designed for treating retinal degenerative diseases [87], but initial results in diseases such as RP did not show any clinically relevant benefit, and since 2013 there have been no novel results in retinal pathologies [88] associated with the use of this technology. The company now seems to be concentrating on glaucoma, although no results have yet been reported.

However, regarding CNTF, there is a question that must be investigated in depth and that is the action of this factor on the glia cells. At least the acute administration of CNTF appears to be related to glial reactivity, which would not be desirable in the context of diseases either of the retina or of the optic nerve [89].

Returning to the CT sponsored by our Eye Institute (NCT03173638), our hypothesis is that ischemic neuropathy can resemble an ischemic stroke, and it should be possible for there to be a series of ganglion cell axons in the so-called penumbra zone. Thus, some of the growth factors released by BMDMSC could “rescue” these fibers, minimizing the damage. Without the presence of growth factors released by mesenchymal stem cells, many of those axons in the “penumbra zone” will die and the functional damage will be greater. A differential fact, in comparison with other CTs, that may be of great relevance is that our cells are from allogeneic sources.

The so-called Stem Cell Ophthalmology Treatment Study (SCOTS) and SCOTS-2 (NCT01920867 and NCT03011541) are especially deserving of interest. They are considered the largest stem cell studies for ocular diseases [90]. The research subjects include dominant optic atrophy and LHON. As in our CT, the stem cell approach is based on the use of BMDMSCs, but in this case the cells are of autologous origin. This is a multicentric study (involving the USA and the United Arab Emirates), and the principal investigators are using mesenchymal stem cells to take advantage of their neuroprotective effects, which have been reported in a variety of animal models of optic nerve damage [91]. Although they are able to differentiate into neurons and glial cells [92], the use of these cells in these CTs is based on their ability to release neurotrophic agents. These neuroprotective properties have been experimentally proven in retinal layers by our group [93,94]. In the SCOTS-2 CT, five patients with LHON reported improvements in visual acuity and peripheral vision. In 2019, in the first SCOTS report, six patients with dominant optic atrophy were included. Five of them experienced visual improvement. The authors speculated that mitochondrial transfer and neuroprotective exosome secretion from mesenchymal stem cells could contribute to this improvement [95]. Nevertheless, these results must be taken with caution, as there was great variability in the treated conditions, including degenerative, ischemic and physical damage of the retina and/or optic nerve. Moreover, the eyes were treated through the injection of BMMSCs, using many different routes of administration—retrobulbar; sub-Tenon and intravenous together, or a combination of retrobulbar, sub-Tenon, intravitreal and intravenous, making the interpretation of their results difficult and creating certain doubts about the quality of the methodology used in the study’s design. Thus, the scientific basis of cell therapy in hereditary optic neuropathies is still under investigation and validation [90].

Regarding the topic of administration routes, those studies that have focused on hematopoietic cell transplantation have obviously used intravenous application, whereas the rest, with two exceptions, have used the intravitreal route. These exceptions are the already mentioned CTs NCT01920867 and NCT03011541, sponsored by the same company, MD Stem Cells (Coral Springs, Florida, USA). This is an interventional, nonmasked, parallel, nonrandomized clinical study, including several retinal conditions and optic nerve diseases (such as glaucoma, optic nerve compression, ischemic optic neuropathy and optic atrophy). The routes for the administration of cells include retrobulbar, subtenon, intravitreal and intravenous routes (alone or as supplements after other routes). The study started in 2016, and the expected date for completion is 2023.

A final reflection can be made on the possibility of using a multimodal therapy for diseases both of the retina and the optic nerve, which are complex and in which perhaps a single therapeutic approach would not work. In a recent paper [96], researchers from Brazil and Florida proposed an interesting combination of gene and cell therapy to increase RGC survival and their axon regrowth. This was an experimental study on a model of optic nerve crush, analyzing the neuroprotective and neuroregenerative potential of pigment epithelium-derived factor (PEDF) gene therapy alone and combined with human mesenchymal stem cell (hMSC) therapy. The authors found a synergistic effect in the combination of gene and cell therapy.

A final point concerns the safety of intravitreal stem cell injections. In a recent paper [97], researchers investigated the vascular outcomes after intravitreal mesenchymal stem cell (MSC) administration in rats, with or without damage to the neurovascular unit (transgenic rats). The authors used rat BMDMSCs and human ADMSCs and found that the intravitreal administration of MSCs induced cataract, retinal vaso-regression, activation of retinal glial cells and an inflammatory response even in normal rat eyes. Our group analyzed the safety of human bone marrow-derived MSCs [98] and these cells were safe and well-tolerated when administered intravitreally at a dose of 15 × 10^6^ cells/mL in pigmented rabbits.

In view of the information analyzed in this review and comparing it with that obtained in our 2016 review [6], it does not appear that there has been much real progress in this field, and it seems that, in the very short term, none of the approaches that are being made in CTs seem to have been transferred to established clinical human treatments.

Ideal cell therapy involves several requisites, such as a source of viable cells, the management of cells under good manufacturing practices (GMPs), reliable delivery methods, long-term survival and functioning of grafted cells without severe adverse effects on the host, and of course a clear objective benefit in terms of the improvement or stabilization of the disease [16].

The main obstacles in this process are derived from the lack of adequacy of the host environment, and the time of use of the cells, which requires the production source close to be close to the clinical place of use. In addition, the short time of release of growth factors by the implanted cells forces us to look for alternatives such as genetically modified cells, which then pose other serious safety problems.

The rescue of RGCs in glaucomatous patients by means of the neuroprotective properties of pluripotent stem cells is a plausible and experimentally proven option. However, the difficulties mentioned above probably influence its very slow development from preclinical research to routine clinical use.

### 4.4. Cell Therapy for Retinal Vascular Diseases

In 2014, Park et al. injected for the first time autologous CD34+ BMMSCs into the vitreous cavities of six patients with retinal vascular occlusion or RD, finding a good safety profile that merits further exploration (NCT01736059) [99]. To date, autologous BMMSCs have been applied by means of intravenous infusion in 34 patients with DR (No. ChiCTR-ONC-16008055; chictr.org. cn). BCVA and central macular thickness, measured with OCT, improved without severe adverse events, mainly in the nonproliferative stage of the disease [100]. Another CT has proposed one intravitreal injection of bone marrow mononuclear stem cells in 30 patients with ischemic retinopathy, including DR with severe loss of retinal capillaries (NCT01518842). This trial is active but not recruiting. Furthermore, a phase I/II, prospective, randomized, sham-controlled, double-masked CT (NCT03981549) is ongoing, aiming to determine whether intravitreal autologous CD34+ stem cell therapy is safe, feasible and potentially beneficial in minimizing or reversing vision loss in eyes with ischemia due to central retinal vein occlusion. Lastly, the combination of CD34+CD45+ cells derived from iPSCs with iPSCs derived from the mesoderm (vascular wall-derived progenitor cells or endothelial colony forming cells—ECFCs) administered into the vitreous cavity is being evaluated in a clinical trial, assessing their potential beneficial effect in preventing microvascular complications in DR (NCT03403699) due to their antioxidative and anti-inflammatory effects.

Finally, a CT intending to evaluate the function of serum exosomal miRNA in the pathogenesis of DR is ongoing (NCT03264976) but not yet recruiting patients [101]. In fact, researchers will try to validate a diagnostic test sequencing these miRNAs and see if they can serve as a prognostic factor. However, according to the available information, it seems that stem cell-derived exosomes may play an important role in RD treatment in the future too. CTs on cell replacement for retinal vascular diseases are presented in Table 6.

## 5. Challenges

Several issues remain to be solved concerning the clinical translation of cell-based therapies, including (1) the ability to enrich for specific retinal subtypes; (2) cell survival; (3) cell delivery, which may need to incorporate a scaffold to induce correct cell polarization, which increases the size of the retinotomy in surgery and, therefore, the chance of severe complications compared to the delivery of isolated cells; (4) the need to induce retinal detachment to perform the subretinal placement of the transplanted cell, which could disrupt the first synapse of the visual pathway and is thought to affect larger areas outside the iatrogenic detachment; (5) the evaluation of the risk of tumor formation caused by undifferentiated stem cells and prolific progenitor cells, which increases when using genome-integrating viruses or gene editing to produce iPSCs because this can cause insertional mutagenesis and unpredictable genetic dysfunction and some transcription factors may have oncogenic properties [5].

The development of surgical techniques for delivering the cells to the right place is one of these challenges. Intravitreal injections have been used, as they are a common procedure in retina patient clinics and are associated with few complications. However, with this route of administration, the concern is that the host retina, mainly the inner limiting membrane, may act as a barrier and prevent the transplanted cells from migrating and integrating into the retinal tissue in the correct location [102]. Therefore, subretinal transplantation is the more commonly used technique when trying to obtain cell replacement, as the cells are delivered to the intended location and therefore better integration and differentiation is observed. However, this is a complex surgical procedure, requiring a skilled retinal surgeon with experience in subretinal surgery, as it has a high risk of surgical complications, including hemorrhage, PVR, graft dislocation and neovascularization [103,104]. If more than one type of cell is needed to restore the natural retinal cell layers, the question will then be whether the layers should be transplanted sequentially or if a retinal complex including the necessary layers would be optimal for the restoration of visual function. When using a cell sheet or an RPE-photoreceptor-scaffold complex, a subretinal approach would be especially necessary, since transplants of this size could not traverse the inner retina, and a purpose-designed microsurgical tool has been proposed to perform these transplants via the suprachoroidal approach, as we have already mentioned [60].

Furthermore, contact with the RPE is essential for photoreceptor cells to properly function. Therefore, new strategies should be found to prevent rosette formation, like transplanting photoreceptors and RPE at the same time [45]. Other attempt to facilitate efficient network formation with host retinal cells is to seed purified photoreceptor cells onto biomaterial sheets and then to transplant them [105].

It is also important to point out that cell survival and transplantation success are determined also by the extent of immune rejection, although we would be working in a relatively immune-privileged site [106]. ESCs do not express major histocompatibility complex (MHC) II and only a low level (although upregulated) of MHC I after transplantation [107]. iPSC-derived cells show less of an immune response [108], but produce an immune response when retroviruses are used to reprogram them [61]. Finally, MHC matching may be beneficial for successful allogeneic stem cell transplantation [109]. All these aspects will be crucial in order to establish the optimal immunosuppression regime for future clinical applications. Moreover, the Center for iPS Cell Research and Application (CiRA) started offering iPSCs stocks for regenerative medicine in 2015, based on the idea that only 10 cell lines carry the three most frequent HLA homologous loci (HLA-A, -B and -DR), thus reducing the possibility of rejection [110]. Therefore, a CT recruited patients suffering from RPE atrophy, who were transplanted with this product without needing systemic immunosuppression, and although one patient showed mild signs of rejection, this was well controlled through the local administration of steroids. However, it resulted in an insufficient number of cells being delivered to the targeted area, which is another problem that should be addressed [111].

On the other hand, future research in regenerative medicine for vascular ischemic retinal diseases must focus on the following issues—(1) whether endothelial precursor cells or MSCs derived from cord blood or pluripotent sources are more pluripotent and therapeutic than adult cells; (2) although adult stem cell therapies are in early I/IIa phase CTd, efficacy and safety results are still pending, and there is a long way to go before their findings can be applied to clinical practice; (3) understanding the interplay between various precursor cells is important in developing the ideal cell therapy for vascular regeneration, since the optimal cell therapy may involve a combination of stem cells or precursor cells; (4) pharmacologic methods aiming to overcome the potential host factors may enhance the regenerative potential of stem cells [112]; (5) understanding the molecular basis for the regenerative effect of stem cells in retinal vascular conditions might shed light on new pharmacologic or genetic approaches to treating retinal vascular disorders and new approaches to enhancing the therapeutic effects of currently available stem cell therapies [113].

As mentioned, the main challenge in OND is the maintenance of RGCs and stimulating the re-growth of their axons [114]. Optic nerve regeneration can be experimentally induced through different approaches, such as by delivering neurotrophic factors, increasing ocular inflammation and manipulating genes targeting growth-related inhibitors, such as phosphatase and tensin homolog (PTEN), Kruppel-like family (KLF) transcription factors and the suppressor of cytokine signaling 3 (SOCS3) [115]. Interestingly, many of these proregenerative pathways are at least indirectly associated with tumor growth, raising concerns about the clinical feasibility of their manipulation [115]. In addition, complex combinatorial approaches are still far from translation.

In 2019, Mesentier et al. [116] showed that intravitreally injected BMMCs promote RGC survival and regeneration after optic nerve crush but RGC survival declined over time. Therefore, one of the challenges is how to maintain the neuroprotective effect over time, especially in diseases in which the etiological treatment is not addressed. The same authors have demonstrated, using an optic nerve crush model, that the intravitreal injection of MSCs sustained RGC neuroprotection and long-distance regeneration, with transient target reconnection, but also with the progressive loss of the axon regenerative effect—an event that is not solely attributed to the clearance of MSCs but also to a limitation of cell therapy alone in achieving permanent neuronal reconnection to its targets. Thus, they suggest that the combination of MSCs or of their secretome with additional therapeutic approaches is more likely to sustain therapeutic effects for a longer time.

The lack of endogenous RGC replacement in mammals differs from what happens in fish and amphibians, which add new RGCs throughout their lifespan, a feature that is thought to arise at least in part from the presence of a specific proneural transcriptional factor, Ascl1, made by retinal Müller glia in cold blooded vertebrates but not by mammalian Müller glia. Three general approaches to replacing RGCs include (i) syngeneic transplantation of adult induced pluripotent stem cells (iPSs) that have been programmed to assume RGC phenotypes, (ii) allogeneic transplantation of RGCs from healthy eyes into host eyes, and (iii) possible reprogramming of endogenous Müller glia into RGCs. Thus, the isolation of RGCs from the retinas of recently deceased humans for transplantation into recipient humans may actually represent a clinically viable strategy for curing otherwise irreversible forms of blindness [10].

As mentioned in the retinal diseases section, another approach could be the possible use of exosomes. Recent evidence has shown that MSCs secrete exosomes, membrane-enclosed vesicles (30–100 nm) containing proteins, mRNA and miRNA, which can be delivered to nearby cells. A recent experimental study in a rat optic nerve crush model demonstrated that exosomes from BMMSCs showed neuroprotective and neuritogenic effects [116]. In this model, BMSC-derived exosomes promoted statistically significant survival of RGCs and regeneration of their axons, while partially preventing RGC axonal loss and RGC dysfunction, opening a treatment possibility as a cell-free therapy for traumatic optic nerve disease, which nevertheless requires further confirmation.

Finally—and since some of the current CTs are directed at the involvement of the optic nerve in multiple sclerosis—it is worth reviewing an experimental approach that may be interesting. Recently, Gramlich et al. [117] aimed to determine the efficacy of MSC therapy on rescuing the visual system in the experimental autoimmune encephalomyelitis (EAE) model of multiple sclerosis (MS). Systemic MSC treatment (intraperitoneally) was found to positively affect RGC function and survival in EAE mice.

In summary, much progress has been made towards translating stem/progenitor cell technology into optimized therapies for retinal and optic nerve diseases, but the road to the clinic will be undeniably long. More defined differentiation protocols are required to improve efficiency and to obtain high-quality enriched retinal cells at the desire state. Notably, insights into human retinal development with the advent of 3D cell culture techniques that mimic in vivo development may help in this regard. Moreover, the genetic modification of stem cells may prove to be a viable approach to generating specific populations of retinal cells that are able to produce some desirable cell products or to be used after correcting a disease-causing mutation. In addition, stem/progenitor cell therapies have already entered early-stage CTs and have demonstrated safety and some indicators of efficacy. Furthermore, the challenge of the immune rejection of transplants needs to be addressed. Currently, stem/progenitor cell therapies for retinal diseases still have some drawbacks, such as inhibition of proliferation and/or differentiation in vitro (with the exception of the RPE) and limited long-term survival and functioning of grafts in vivo. Despite these challenges, stem/progenitor cells represent the most promising strategy for retinal and optic nerve disease treatment in the near future, as therapeutic strategies assisted by gene techniques, neuroprotective compounds and artificial devices can be applied to fulfil clinical needs. Finally, the collaboration of various experts in engineering, cell biology, genetics and clinical medicine is essential for the development of successful cell therapies.

## 6. Conclusions

Much progress has been made towards translating stem/progenitor cell technology into optimized therapies for retinal and optic nerve diseases demonstrating safety and efficacy. However, scientists need to work in more defined differentiation protocols and immune rejection of transplants, as well as provide insights into human retinal development and genetic modification of stem cells.

## Figures and Tables

**Table 1:** Stem cell sources and their potential for the treatment of retinal and optic nerve diseases.

Stem Cell Source	Main Advantages and Disadvantages	Cell Type	Potential Applications
	**Retinal Progenitor Cells**		
Fetal stem cells	Simple accessibility, safety and effectiveness Shortage of sufficient donor cells Limited proliferative capacity Restricted ability to differentiate into specific types of cells Relatively low risk of immune rejection and tumorigenesis	Retinal progenitor cells (RPCs)	Paracrine neuroprotection Exogenous cell replacement
		Cortical progenitor cells (CPCs)	Paracrine neuroprotection
	**Pluripotent Stem Cells**		
Human embryonic stem cells	Ability to differentiate into photoreceptors under certain circumstances, but presenting difficulties in obtaining a specific targeted cell type Shortage of sufficient donor cells Limited proliferative capacity Restricted ability to differentiate into specific targeted cells Potential of tumor formation Requires immunosuppressive treatment increasing risks and burden Ethical concerns	Human embryonic stem cell derived retinal pigment epitheliums (hESC-RPE)	Exogenous cell replacement Non-cell-based therapy screening
Adult induced pluripotent stem cells	Able to provide large number of cells for treatments Low risk of immune reaction (autologous) Ameliorate the ethical issues of hESCs Low differentiation efficiency Relatively high risk of gene mutation	Adult induced pluripotent stem cells (iPSC)	Exogenous cell replacement Disease modeling Non-cell-based therapy screening
	**Multipotent Stem Cells**		
Mesenchymal stem cells	Able to provide large number of cells for treatments ADRCs obtained in less invasive procedures and higher immunomodulatory capacity than BMSCs Anti-inflammatory immunosuppressive antiangiogenic and antiapoptotic or neuroprotective effects Ability to differentiate into damaged cells Low rate of cell migration and differentiation Reported to differentiate into photoreceptors and retinal pigment epithelial (RPE) cells	Bone marrow-derived stem cells (BMSCs)	Paracrine neuroprotection
		Adipose-derived stem cells (ADRCs)	Paracrine neuroprotection
	Higher antiapoptotic effect Strong rescue effect on retinal function Potential RPE cell differentiation capacity	Human umbilical multipotent stem cells retrieved from donor umbilical cords (hUTSCs)	Paracrine neuroprotection
Other sources		Ciliary epithelium-derived stem cells (CESCs)	Exogenous cell replacement Endogenous cell replacement?
		Cells extracted from the adult human RPE, obtained from eye banks and activated in vitro into a stem cell state (RPESCs)	Exogenous cell replacement Endogenous cell replacement?
		Reprogrammed endogenous Müller glia into RGCs (hMSCs)	Exogenous cell replacement Endogenous cell replacement?

**Table 2:** RPE replacement clinical trials.

Reference	Cell Type	Title	Disease	Administration Procedure	Status
NCT01226628 Phase I/IIa Study	Human umbilical multipotent stem cells retrieved from donor umbilical cords (hUTSCs)	A Safety Study of CNTO 2476 in Patients With Age-Related Macular Degeneration	Geographic atrophy due to age-related macular Degeneration	Subretinal with the iTrack Model 275 micro catheter	Completed
NCT01914913 Phase I/II Study	Autologous bone marrow derived mono nuclear stem cells (BMMNCs)	Clinical Study to Evaluate Safety and Efficacy of BMMNC in Retinitis Pigmentosa	Retinitis pigmentosa	Intravitreal	Unknown
NCT02280135 Phase I Study	Autologous bone marrow stem cells	Clinical Trial of Intravitreal Injection of Autologous Bone Marrow Stem Cells in Patients With Retinitis Pigmentosa	Retinitis pigmentosa	Intravitreal	Completed
NCT01560715 Phase II Study	Autologous bone marrow stem cells	Autologous Bone Marrow-Derived Stem Cells Transplantation For Retinitis Pigmentosa	Retinitis pigmentosa	Intravitreal	Completed
NCT01531348 Phase I Study	Human bone marrow-derived mesenchymal stem cells	Feasibility and Safety of Human Bone Marrow-derived Mesenchymal Stem Cells by Intravitreal Injection in Patients With Retinitis Pigmentosa	Retinitis pigmentosa	Subretinal	Unknown
NCT02016508 Phase I/II Study	Autologous bone marrow derived stem cells	Safety Study of Use of Autologous Bone Marrow Derived Stem Cell in Treatment of Age Related Macular Degeneration	Age-related macular degeneration	Intravitreal	Unknown
NCT03944239 Phase I Study	Retinal pigment epithelial (RPE) cells derived from human embryonic stem cells (hESC)	Safety and Efficacy of Subretinal Transplantation of Clinical Human Embryonic Stem Cell Derived Retinal Pigment Epitheliums in Treatment of Retinitis Pigmentosa	Retinitis pigmentosa	Subretinal	Recruiting
NCT02749734 Phase I/II Study	Human embryonic stem cell derived retinal pigment epitheliums (hESC-RPE)	Clinical Study of Subretinal Transplantation of Human Embryo Stem Cell Derived Retinal Pigment Epitheliums in Treatment of Macular Degeneration Diseases	Macular degeneration and Stargardt’s macular dystrophy	Subretinal	Unknown
NCT03046407 Phase I/II Study	Human embryonic stem cell derived retinal pigment epitheliums (hESC-RPE)	Treatment of Dry Age Related Macular Degeneration Disease With Retinal Pigment Epithelium Derived From Human Embryonic Stem Cells	Dry age-related macular degeneration	Subretinal	Unknown
NCT03167203 Phase I/II Study	Human embryonic stem cell derived retinal pigment epitheliums (hESC-RPE)	A Safety Surveillance Study in Subjects With Macular Degenerative Disease Treated With Human Embryonic Stem Cell-derived Retinal Pigment Epithelial Cell Therapy	Macular degenerative disease	Subretinal	Enrolling by invitation
NCT02941991 Follow up Study	Human embryonic stem cell derived retinal pigment epitheliums (hESC-RPE)	A Follow up Study to Determine the Safety and Tolerability of Sub-retinal Transplantation of Human Embryonic Stem Cell Derived Retinal Pigmented Epithelial (hESC-RPE) Cells in Patients With Stargardt’s Macular Dystrophy (SMD)	Stargardt’s macular dystrophy	Biological: hESC-RPE	Completed
NCT02903576 Phase I/II Study	Procedure: injection of hESC-RPE in suspension Procedure: injection hESC-RPE seeded in a substrate	Stem Cell Therapy for Outer Retinal Degenerations	Age-related macular degeneration Stargardt’s disease Exudative age-related macular degeneration	Subretinal	Completed
NCT01345006 Phase I/II Study	Human embryonic stem cell derived retinal pigment epithelium cells Biological: (MA09-hRPE)	Sub-retinal Transplantation of hESC Derived RPE(MA09-hRPE) Cells in Patients With Stargardt’s Macular Dystrophy	Stargardt’s macular dystrophy	Subretinal	Completed
NCT01469832 Phase I/II Study	Human embryonic stem cell derived retinal pigment epithelium cells Biological: (MA09-hRPE)	Safety and Tolerability of Sub-retinal Transplantation of Human Embryonic Stem Cell Derived Retinal Pigmented Epithelial (hESC-RPE) Cells in Patients With Stargardt’s Macular Dystrophy (SMD)	Stargardt’s macular dystrophy	Subretinal	Completed
NCT01344993 Phase I/II Study	Human embryonic stem cell derived retinal pigment epithelium cells Biological: (MA09-hRPE)	Safety and Tolerability of Sub-retinal Transplantation of hESC Derived RPE (MA09-hRPE) Cells in Patients With Advanced Dry Age Related Macular Degeneration	Dry age-related macular degeneration	Subretinal	Completed
NCT01344993 Phase I/II Study	Human embryonic stem cell derived retinal pigment epithelium cells Biological: (MA09-hRPE)	Safety and Tolerability of Sub-retinal Transplantation of hESC Derived RPE (MA09-hRPE) Cells in Patients With Advanced Dry Age Related Macular Degeneration	Dry age-related macular degeneration	Subretinal	Completed
NCT02463344 Phase I/II Study	Human embryonic stem cell derived retinal pigment epithelium cells Biological: (MA09-hRPE)	Long Term Follow Up of Sub-retinal Transplantation of hESC Derived RPE Cells in Patients With AMD	Age-related macular degeneration	Subretinal	Completed
NCT01345006 Phase I/II Study	Human embryonic stem cell derived retinal pigment epithelium cells Biological: (MA09-hRPE)	Sub-retinal Transplantation of hESC Derived RPE(MA09-hRPE)Cells in Patients With Stargardt’s Macular Dystrophy	Stargardt’s macular dystrophy	Subretinal	Completed
NCT02445612 Long term follow up	Human embryonic stem cell derived retinal pigment epithelium cells Biological: (MA09-hRPE)	Long Term Follow Up of Sub-retinal Transplantation of hESC Derived RPE Cells in Stargardt Macular Dystrophy Patients	Stargardt’s macular dystrophy	Subretinal	Completed
NCT01469832 Phase I/II Study	Human embryonic stem cell derived retinal pigment epithelium cells Biological: (MA09-hRPE)	Safety and Tolerability of Sub-retinal Transplantation of Human Embryonic Stem Cell Derived Retinal Pigmented Epithelial (hESC-RPE) Cells in Patients With Stargardt’s Macular Dystrophy (SMD)	Stargardt’s macular dystrophy	Subretinal	Completed
NCT01625559 Phase I/II Study	Human embryonic stem cell derived retinal pigment epithelium cells Biological: (MA09-hRPE)	Safety and Tolerability of MA09-hRPE Cells in Patients With Stargardt’s Macular Dystrophy(SMD)	Stargardt’s macular dystrophy	Subretinal	Unknown
NCT01674829 Phase I/II Study	Human embryonic stem cell derived retinal pigment epithelium cells Biological: (MA09-hRPE)	A Phase I/IIa, Open-Label, Single-Center, Prospective Study to Determine the Safety and Tolerability of Sub-retinal Transplantation of Human Embryonic Stem Cell Derived Retinal Pigmented Epithelial(MA09-hRPE) Cells in Patients With Advanced Dry Age-related Macular Degeneration(AMD)	Dry age-related macular degeneration	Subretinal	Active, not recruiting
NCT02286089 Phase I/II Study	Retinal pigment epithelial (RPE) cells derived from human embryonic stem cells (hESC) Biological: OpRegen: cell suspension either in ophthalmic Balanced Salt Solution Plus (BSS Plus) or in CryoStor^®^ 5 (Thaw-and-Inject, TAI)	Safety and Efficacy Study of OpRegen for Treatment of Advanced Dry-Form Age-Related Macular Degeneration	Age-related macular degeneration	Subretinal	Active, not recruiting
NCT03963154 Phase I/II Study	Human embryonic stem cell derived retinal pigment epithelium (RPE) Investigational Medicinal Product: ISTEM-01	Interventional Study of Implantation of hESC-derived RPE in Patients With RP Due to Monogenic Mutation	Retinitis pigmentosa	Subretinal	Recruiting
NCT02590692 Phase I/II Study	Human embryonic stem cell-derived RPE cells Biological: CPCB-RPE1	Study of Subretinal Implantation of Human Embryonic Stem Cell-Derived RPE Cells in Advanced Dry AMD	Dry macular degeneration Geographic atrophy	Subretinal	Active, not recruiting
NCT03102138 Safety follow up Study	Human embryonic stem cell-derived RPE cells Biological: PF-05206388	Retinal Pigment Epithelium Safety Study For Patients In B4711001	Age-related macular degeneration	Intravitreal	Active, not recruiting
NCT01691261 Phase I Study	Human embryonic stem cell derived retinal pigment epithelium (RPE) living tissue equivalent Biological: PF-05206388: monolayer of RPE cells immobilized on a polyester membrane	A Study Of Implantation Of Retinal Pigment Epithelium In Subjects With Acute Wet Age Related Macular Degeneration	Age-related macular degeneration	Intraocular	Active, not recruiting
NCT02464956 Feasibility of production of these cells	Induced pluripotent stem cell (iPSC)-derived RPE cells from a patient’s own skin or blood	Production of iPSC Derived RPE Cells for Transplantation in AMD	Age-related macular degeneration	None	Unknown

**Table 3:** Photoreceptor replacement clinical trials.

Reference	Cell Type	Title	Disease	Administration Procedure	Status
NCT02464436 Phase I/IIa Study	Human retinal progenitor cells (hRPC)	Safety and Tolerability of hRPC in Retinitis Pigmentosa	Retinitis pigmentosa	Subretinal	Recruiting
NCT01068561 Phase I Study	Autologous bone marrow-derived stem cells	Autologous Bone Marrow-Derived Stem Cells Transplantation For Retinitis Pigmentosa	Retinitis pigmentosa	Intravitreal	Completed
NCT01560715 Phase II Study	Autologous bone marrow stem cells	Autologous Bone Marrow-Derived Stem Cells Transplantation For Retinitis Pigmentosa	Retinitis pigmentosa	Intravitreal	Completed
NCT01518127 Phase I/II Study	Autologous bone marrow stem cells	Intravitreal Bone Marrow-Derived Stem Cells in Patients With Macular Degeneration	Age-related macular degeneration and Stargartd	Intravitreal	Completed
NCT03437759 Phase I Study	Biological: exosomes derived from mesenchymal stem cells (MSC-Exo)	MSC-Exos Promote Healing of MHs	Macular holes	Intravitreal during a vitrectomy and the aid of endotamponades	Active, not recruiting
NCT03853252 Not applicable (proof of concept)	Autologous skin biopsy to get cells from choroideremia patients	iPS Cells of Patients for Models of Retinal Dystrophies	Retinal dystrophies: choroideremia	Other: create cell models of disease	Recruiting

**Table 4:** Optic nerve regeneration: failed cell therapy clinical trials for optic nerve disorders.

Reference	Disease	Cell Type	Administration Route	Study Start Date	Status
NCT01364246 Phase I/II Study	Multiple sclerosis and neuromyelitis optica	Human umbilical multipotent stem cells retrieved from donor umbilical cords (hUTSCs)	Transplantation	January 2010	Unknown
NCT01834079 Phase I/II Study	Optic nerve atrophy	Autologous bone marrow derived stem cells	Intrathecal injection	September 2014	Unknown
NCT02249676 Phase II Study	Progressive and refractory neuromyelitis optica spectrum disorders	Autologous mesenchymal stem cells	Intravenous infusion of MSC a day-case 2.0 × 106 cells/kg	January 2013	Unknown
NCT03605238 Phase I Study	Relapsed and/or refractory AQP4-IgG seropositive neuromyelitis optica spectrum disorders	CD19/CD20 tanCAR T Cells	Intravenous infusion	August 2018	Withdrawn
NCT02976441 Phase I Study	High grade gliomas	Autologous stem cell collection	Stem cell intravenous infusion prior chemoradiation and reinfused back after treatment	January 2017	Withdrawn
NCT02144103 Phase I/II Study	Retinal degeneration and primary open-angle glaucoma	Autologous adipose-derived regenerative cells (ADRC)	Subtenon	May 2014	Unknown
NTC 01339455 Phase I/II Study	Neuromyelitis optica	Autologous hematopoietic stem cells	Intravenous infusion	April 2011	Terminated (recruitment failure)

**Table 5:** Optic nerve regeneration: cell therapy clinical trials for optic nerve disorders.

Reference	Disease	Cell Type	Administration Route	Sponsor	Study Start Date	Status
NTC 02638714 Phase I/II Study	Optic nerve atrophy	Autologous bone marrow CD 34+, 133+, and 271+ stem cells	No site declared	Stem Cells Arabia	April 2013	Recruiting
NTC 03173638 Phase II Study	Acute ischemic optic neuropathy nonarteritic	Allogenic mesenchymal stem (MSV) cells from bone marrow	Intravitreal injection	IOBA, Spain	March 2018	Recruiting
NCT 022836771 Phase I Study	Neuromyelitis optica	Tolerogenic dendritic cells loaded with myelin peptides	Intravenous administration	Hospital Clinic of Barcelona, Spain	September 2015	Completed
NTC 01920867 Phase (n/a)	Various ocular diseases including optic neuritis	Bone marrow derived stem cells (BMSC). Study I	Injections of BMSC retrobulbar, subtenon and intravenous	MD Stem Cells, USA	August 2012	Enrolling by invitation
NTC 03011541 Phase (n/a)	Various ocular diseases including optic neuropathy Nonarteritic ischemic optic neuropathy Optic atrophy, optic nerve disease, glaucoma, Leber hereditary optic neuropathy	Bone marrow derived stem cells (BMSC). Study II	Injections of BMSC retrobulbar, subtenon and intravenous	MD Stem Cells, USA	January 2016	Recruiting
NTC 00787722 Phase I/II Study	Devic neuromyelitis	High dose immunosuppressive therapy with hematopoietic stem cells transplantation	Intravenous infusion	Northwestern University, USA	October 2009	Completed
NTC 00716066 Phase II Study	Neurologic autoimmune diseases, including neuromyelitis optica	High dose immunosuppressive therapy with autologous hematopoietic stem cell transplantation	Intravenous infusion	Fred Hutchinson Cancer Research Center National Cancer Institute, USA	June 2008	Recruiting
NTC 04577300 Phase II Study	Glaucoma	Dual NT-501 CNTF encapsulated cell therapy	Intravitreal NT-501 implants	Stanford University, USA	October 2020	Not yet recruiting
NTC 02862938 Phase II Study	Glaucoma	NT-501 CNTF encapsulated cell therapy	Intravitreal NT-501 implants	Stanford University, USA	August 2016	Active, not recruiting
NTC 02330978 Phase I Study	Glaucoma	Autologous bone marrow-derived mesenchymal stem cell	Intravitreal	University of Sao Paulo, Brazil	July 2019	Completed

(n/a): not applicable; (CNTF): soluble ciliary neurotrophic factor.

**Table 6:** Cell replacement clinical trials for retinal vascular diseases.

Reference	Cell Type	Title	Disease	Administration Procedure	Status
NCT01518842 Not applicable	Bone marrow stem cells	Effect of Intravitreal Bone Marrow Stem Cells on Ischemic Retinopathy (RetinaCell)	Ischemic retinopathy, including diabetic retinopathy with severe loss of retinal capillaries	Intravitreal	Unknown
NCT01736059 Phase I Study	CD34+ autologous adult bone marrow stem cells intravitreal	Clinical Trial of Autologous Intravitreal Bone-marrow CD34+ Stem Cells for Retinopathy	Non-exudative age-related macular degeneration Diabetic retinopathy Retina vein occlusion Retinitis pigmentosa hereditary macular degeneration	Intravitreal	Enrolling by invitation
NCT03981549 Phase I/II Study	CD34+ autologous bone marrow stem cells versus sham therapy	Treatment of Central Retinal Vein Occlusion Using Stem Cells Study (TRUST)	Central retinal vein occlusion	Intravitreal	Recruiting
NCT03403699 Not applicable	Combination of CD34+CD45+ cells derived from human inducible pluripotent stem cells (iPSCs) with iPSCs derived from the mesoderm: vascular wall-derived progenitor cells or endothelial colony forming cells (ECFCs) subset (SSEA5-KNA+)	Human iPSC for Repair of Vasodegenerative Vessels in Diabetic Retinopathy	Diabetes complications Diabetic retinopathy	Others: to test if the hiPSC-derived-mesoderm subset (SSEA5-KNA+) can revascularize vasodegenerative capillaries and if their reparative action can be enhanced by coinjection of CD34+CD45+ cells intravitreally.	Recruiting
NCT03264976 Not applicable	None	Role of the Serum Exosomal miRNA in Diabetic Retinopathy (DR)	Diabetic retinopathy	Validation of a diagnostic test based on exosomal miRNAs in serum samples that will be sequenced	Not yet recruiting

## Data Availability

Not applicable.

## References

[1] de Jong P.T. (2006). Age-related macular degeneration. N. Engl. J. Med..

[2] Ferrari S., Di Iorio E., Barbaro V., Ponzin D., Sorrentino F.S., Parmeggiani F. (2011). Retinitis pigmentosa: Genes and disease mechanisms. Curr. Genom..

[3] Osborne N.N., Casson R.J., Wood J.P., Chidlow G., Graham M., Melena J. (2004). Retinal ischemia: Mechanisms of damage and potential therapeutic strategies. Prog. Retin Eye Res..

[4] Barber A. (2003). A new view of diabetic retinopathy: A neurodegenerative disease of the eye. Prog. Neuro-Psychopharmacol. Biol. Psychiatry.

[5] Zarbin M. (2019). Cell-Based Therapy for Retinal Disease: The New Frontier. Methods Mol. Biol..

[6] Labrador-Velandia S., Alonso-Alonso M.L., Alvarez-Sanchez S., González-Zamora J., Carretero-Barrio I., Pastor J.C., Fernandez-Bueno I., Srivastava G.K. (2016). Mesenchymal stem cell therapy in retinal and optic nerve diseases: An update of clinical trials. World J. Stem Cells.

[7] Fu L., Kwok S.S., Chan Y.K., Lai J.S., Pan W., Nie L., Shih K.C. (2019). Therapeutic Strategies for Attenuation of Retinal Ganglion Cell Injury in Optic Neuropathies: Concepts in Translational Research and Therapeutic Implications. BioMed Res. Int..

[8] DeBusk A., Moster M.L. (2018). Gene therapy in optic nerve disease. Curr. Opin. Ophthalmol..

[9] Moore D.L., Goldberg J.L. (2010). Four steps to optic nerve regeneration. J. Neuroophthalmol..

[10] Laha B., Stafford B.K., Huberman A.D. (2017). Regenerating optic pathways from the eye to the brain. Science.

[11] Chun B.Y., Cestari D.M. (2017). Advances in experimental optic nerve regeneration. Curr. Opin. Ophthalmol..

[12] Cenni M.C., Bonfanti L., Martinou J.C., Ratto G.M., Strettoi E., Maffei L. (1996). Long-term survival of retinal ganglion cells following optic nerve section in adult bcl-2 transgenic mice. Eur. J. Neurosci..

[13] Bonfanti L., Strettoi E., Chierzi S., Cenni M.C., Liu X.H., Martinou J.-C., Maffei L., Rabacchi S.A. (1996). Protection of retinal ganglion cells from natural and axotomy-induced cell death in neonatal transgenic mice overexpressing bcl-2. J. Neurosci..

[14] Maes M.E., Schlamp C.L., Nickells R.W. (2017). BAX to basics: How the BCL2 gene family controls the death of retinal ganglion cells. Prog. Retin Eye Res..

[15] Puertas-Neyra K., Usategui-Martín R., Coco R.M., Fernandez-Bueno I. (2020). Intravitreal stem cell paracrine properties as a potential neuroprotective therapy for retinal photoreceptor neurodegenerative diseases. Neural Regen. Res..

[16] Shen Y. (2020). Stem cell therapies for retinal diseases: From bench to bedside. J. Mol. Med..

[17] Huang S.S. (2020). Future vision 2020 and beyond. 5 critical trends in eye research. Asia Pac. J. Ophthalmol..

[18] Kannabiran C., Mariappan I. (2018). Therapeutic avenues for hereditary forms of retinal blindness. J. Genet..

[19] Salero E., Blenkinsop T.A., Corneo B., Harris A., Rabin D., Stern J.H., Temple S. (2012). Adult human RPE can be activated into a multipotent stem cell that produces mesenchymal derivatives. Cell Stem Cell.

[20] Wang Y., Tang Z., Gu P. (2020). Stem/progenitor cell-based transplantation for retinal degeneration: A review of clinical trials. Cell Death Dis..

[21] Tang Z., Zhang Y., Wang Y., Zhang D., Shen B., Luo M., Gu P. (2017). Progress of stem/progenitor cell-based therapy for retinal degeneration. J. Transl. Med..

[22] Caplan A., Deniis J. (2006). Mesenchymal stem cells as trophic mediators. J. Cell Biochem..

[23] Chamberlian G., Fox J., Ashton B., Middleton J. (2007). Concise review: Mesenchymal stem cells: Their phenotype, differentiation capacity, immunological features, and potential for homing. Stem Cells.

[24] Megaw R., Dhillon B. (2014). Stem cell therapies in the management of diabetic retinopathy. Curr. Diab. Rep..

[25] Alvarez-Palomo A.B., McLenachan S., Chen F.K., Da Cruz L., Dilley R.J., Requena J., Lucas M., Lucas A., Drukker M., Edel M.J. (2015). Prospects for clinical use of IPCS. Fibrogenesis Tissue Repair.

[26] Mandai M., Kurimoto Y., Takahashi M. (2017). Comment: Autologous induced stem-cell-derived retinal cells for macular degeneration. N. Engl. J. Med..

[27] Nakano T., Ando S., Takata N., Kawada M., Muguruma K., Sekiguchi K., Saito K., Yonemura S., Eiraku M., Sasai K. (2012). Self-formation of optic cups and storable stratified neural retina from human ESCs. Cell Stem Cell.

[28] Reichman S., Slembrouck A., Gagliardi G., Chaffiol A., Terray A., Nanteau C., Potey A., Belle M., Rabesandratana O., Duebel J. (2017). Generation of Storable Retinal Organoids and Retinal Pigmented Epithelium from Adherent Human iPS Cells in Xeno-Free and Feeder-Free Conditions. Stem Cells.

[29] Tanaka T., Yokoi T., Tamalu F., Watanabe S.-I., Nishina S., Azuma N. (2015). Generation of retinal ganglion cells with functional axons from human induced pluripotent stem cells. Sci. Rep..

[30] Kim H., Kim J.J., Yoon Y.S. (2012). Emerging therapy for diabetic neuropathy: Cell therapy targeting vessels and nerves. Endocr. Metab. Immune Disord. Drug Targets.

[31] Park S.S. (2016). Cell Therapy Applications for Retinal Vascular Diseases: Diabetic Retinopathy and Retinal Vein Occlusion. Investig. Ophthalmol. Vis. Sci..

[32] Asahara T., Murohara T., Sullivan A., Silver M., van der Zee R., Li T., Witzenbichler B., Schatteman G., Isner J.M. (1997). Isolation of putative progenitor endothelial cells for angiogenesis. Science.

[33] Mackie A.R., Losordo D.W. (2011). CD34 positive stem cells in the treatment of heart and vascular disease in human beings. Tex. Heart Inst. J..

[34] Caballero S., Sengupta N., Afzal A., Chang K.H., Li Calzi S., Guberski D.L., Kern T.S., Grant M.B. (2007). Ischemic vascular damage can be repaired by healthy, but not diabetic, endothelial progenitor cells. Diabetes.

[35] Goldenberg-Cohen N., Avraham-Lubin B.C., Sadikov T., Askenasy N. (2014). Effect of co-administration of neuronal growth factors on neuroglial differentiation of bone marrow-derived stem cells in the ischemic retina. Investig. Ophthalmol. Vis. Sci..

[36] Medina R.J., O’Neill C.L., Humphreys M.W., Gardiner T.A., Stitt A.W. (2010). Outgrowth endothelial cells: Characterization and their potential for reversing ischemic retinopathy. Investig. Ophthalmol. Vis. Sci..

[37] Mendel T.A., Clabough E.B., Kao D.S., Demidova-Rice T.N., Durham J.T., Zotter B.C., Seaman S.A., Cronk S.M., Rakoczy E.P., Katz A.J. (2013). Pericytes derived from adipose-derived stem cells protect against retinal vasculopathy. PLoS ONE.

[38] Prasain N., Lii M.R., Vemula S., Meador J.L., Yoshimoto M., Ferkowicz M.J., Fett A., Gupta M., Rapp B.M., Saadatzadeh M.R. (2014). Differentiation of human pluripotent stem cells to cells similar to cord-blood endothelial colony-forming cells. Nat. Biotechnol..

[39] Park T.S., Bhutto I., Zimmerlin L., Huo J.S., Nagaria P., Miller D., Rufaihah A.J., Talbot C., Aguilar J., Grebe R. (2014). Vascular progenitors from cord blood-derived induced pluripotent stem cells possess augmented capacity for regenerating ischemic retinal vasculature. Circulation.

[40] Moisseiev E., Anderson J.D., Oltjen S., Goswami M., Zawadzki R.J., Nolta J.A., Park S.S. (2017). Protective Effect of Intravitreal Administration of Exosomes Derived from Mesenchymal Stem Cells on Retinal Ischemia. Curr. Eye Res..

[41] Safwat A., Sabry D., Ragiae A., Amer E., Mahmoud R.H., Shamardan R.M. (2018). Adipose mesenchymal stem cells-derived exosomes attenuate retina degeneration of streptozotocin-induced diabetes in rabbits. J. Circ. Biomark..

[42] Alexander P., Thomson H.A., Luff A.J., Lotery A.J. (2015). Retinal pigment epithelium transplantation: Concepts, challenges, and future prospects. Eye.

[43] Binder S., Stolba U., Krebs I., Kellner L., Jahn C., Feichtinger H., Povelka M., Frohner U., Kruger A., Hilgers R.D. (2002). Transplantation of autologous retinal pigment epithelium in eyes with foveal neovascularization resulting from age-related macular degeneration: A pilot study. Am. J. Ophthalmol..

[44] Li L.X., Turner J.E. (1988). Inherited retinal dystrophy in the RCS rat: Prevention of photoreceptor degeneration by pigment epithelial cell transplantation. Exp. Eye Res..

[45] Uyama H., Mandai M., Takahashi M. (2020). Stem Cell-Based Therapies for Retinal Degenerative Diseases: Current Challenges in the Establishment of New Treatment Strategies. Dev. Growth Differ..

[46] Satarian L., Nourinia R., Safi S., Kanavi M.R., Jarughi N., Daftarian N., Arab L., Aghdami N., Ahmadieh H., Baharvand H. (2017). Intravitreal injection of bone marrow mesenchymal stem cells in patients with advanced retinitis pigmentosa; a safety study. J. Ophthalmic Vis. Res..

[47] Egypt Al-Azhar University Safety Study of Use of Autologuous Bone Marrow Derived Stem Cell in Treatment of Age Related Macular Degeneration. https://clinicaltrials.gov/ct2/show/study/NCT02016508.

[48] Schwartz S.D., Hubschman J.-P., Heilwell G., Franco-Cardenas V., Pan C.K., Ostrick R.M., Mickunas E., Gay R., Klimanskaya I., Lanza R. (2012). Embryonic stem cell trials for macular degeneration: A preliminary report. Lancet.

[49] Schwartz S., Regillo C., Lam B., Eliott D., Rosenfeld P., Gregori N., Hubschman J.-P., Davis J., Heilwell G., Spirn M. (2015). Human embryonic stem cell-derived retinal pigment epithelium in patients with age related macular degeneration and Stargardt’s macular dystrophy: Follow-up of two open-label phase 1/2 studies. Lancet.

[50] Mehat M.S., Sundaram V., Ripamonti C. (2018). Transplantation of Human Embryonic Stem Cell-Derived Retinal Pigment Epithelial Cells in Macular Degeneration. Ophthalmology.

[51] Song W.K., Park K.M., Kim H.J., Lee J.H., Choi J., Chong S.Y., Shim S.H., Del Priore L.V., Lanza R. (2015). Treatment of macular degeneration using embryonic stem cell-derived retinal pigment epithelium: Preliminary results in Asian patients. Stem Cell Rep..

[52] Liu Y., Xu H.W., Wang L., Li S.Y., Zhao C.J., Hao J., Li Q.Y., Zhao T.T., Wu W., Wang Y. (2018). Human embryonic stem cell-derived retinal pigment epithelium transplants as a potential treatment for wet age-related macular degeneration. Cell Discov..

[53] Mandai M., Watanabe A., Kurimoto Y., Hirami Y., Morinaga C., Daimon T., Fujihara M., Akimaru H., Sakai N., Shibata Y. (2017). Autologous induced stem-cell–derived retinal cells for macular degeneration. N. Engl. J. Med..

[54] Production of iPSC Derived RPE Cells for Transplantation in AMD. ClinicalTrials.gov. Identifier: NCT02464956. Last Updated: 8 June 2015. NCT02464956.

[55] Xu H., Wang B., Ono M., Kagita A., Fujii K., Sasakawa N., Ueda T., Gee P., Nishikawa M., Nomura M. (2019). Targeted Disruption of HLA Genes via CRISPR-Cas9 Generates iPSCs with Enhanced Immune Compatibility. Cell Stem Cell.

[56] Maeda T., Lee M.J., Palczewska G., Marsili S., Tesar P.J., Palczewski K., Takahashi M., Maeda A. (2013). Retinal pigmented epithelial cells obtained from human induced pluripotent stem cells possess functional visual cycle enzymes in vitro and in vivo. J. Biol. Chem..

[57] Kamao H., Mandai M., Okamoto S., Sakai N., Suga A., Sugita S., Kiryu J., Takahashi M. (2014). Characterization of Human Induced Pluripotent Stem Cell-Derived Retinal Pigment Epithelium Cell Sheets Aiming for Clinical Application. Stem Cell Rep..

[58] Ben M’Barek K., Habeler W., Monville C. (2018). Stem Cell-Based RPE Therapy for Retinal Diseases: Engineering 3D Tissues Amenable for Regenerative Medicine. Adv. Exp. Med. Biol..

[59] Kashani A.H., Uang J., Mert M., Rahhal F., Chan C., Avery R.L., Dugel P., Chen S., Lebkowski J., Clegg D.O. (2020). Surgical Method for Implantation of a Biosynthetic Retinal Pigment Epithelium Monolayer for Geographic Atrophy: Experience from a Phase 1/2a Study. Ophthalmol. Retin..

[60] da Cruz L., Fynes K., Georgiadis O., Kerby J., Luo Y.H., Ahmado A., Vernon A., Daniels J.T., Nommiste B., Hasan S.M. (2018). Phase 1 clinical study of an embryonic stem cell-derived retinal pigment epithelium patch in age-related macular degeneration. Nat. Biotechnol..

[61] Zhao C., Wang Q., Temple S. (2017). Stem cell therapies for retinal diseases: Recapitulating development to replace degenerated cells. Development.

[62] Zhu D., Deng X., Spee C., Sonoda S., Hsieh C.L., Barron E., Pera M., Hinton D.R. (2011). Polarized secretion of PEDF from human embryonic stem cell-derived RPE promotes retinal progenitor cell survival. Investig. Ophthalmol. Vis. Sci..

[63] Radtke N.D., Aramant R.B., Seiler M.J., Petry H.M., Pidwell D. (2004). Vision change after sheet transplant of fetal retina with retinal pigment epithelium to a patient with retinitis pigmentosa. Arch. Ophthalmol..

[64] Aramant R.B., Seiler M.J. (2004). Progress in retinal sheet transplantation. Prog. Retin Eye Res..

[65] Cordero A., West E.L., Pearson R.A., Duran Y., Carvalho L.S., Chu C.J., Naeem A., Blackford S.J.I., Georgiadis A., Lakowski J. (2013). Photoreceptor precursors derived from three-dimensional embryonic stem cell cultures integrate and mature within adult degenerate retina. Nat. Biotechnol..

[66] Klassen H.J., Ng T.F., Kurimoto Y., Kirov I., Shatos M., Coffey P., Young M.J. (2004). Multipotent retinal progenitors express developmental markers, differentiate into retinal neurons, and preserve light-mediated behavior. Investig. Ophthalmol. Vis. Sci..

[67] Lamba D.A., Gust J., Reh T.A. (2009). Transplantation of Human Embryonic Stem Cell-Derived Photoreceptors Restores Some Visual Function in Crx-Deficient Mice. Cell Stem Cell.

[68] Tucker B.A., Park I.H., Qi S.D., Klassen H.J., Jiang C., Yao J., Redenti S., Daley G.Q., Young M.J. (2011). Transplantation of adult mouse iPS cell-derived photoreceptor precursors restores retinal structure and function in degenerative mice. PLoS ONE.

[69] Homma K., Okamoto S., Mandai M., Gotoh N., Rajasimha H.K., Chang Y.S., Chen S., Li W., Cogliati T., Swaroop A. (2013). Developing rods transplanted into the degenerating retina of Crx-knockout mice exhibit neural activity similar to native photoreceptors. Stem Cells.

[70] Santos-Ferreira T., Völkner M., Borsch O., Haas J., Cimalla P., Vasudevan P., Carmeliet P., Corbeil D., Michalakis S., Koch E. (2016). Stem Cell-Derived Photoreceptor Transplants Differentially Integrate Into Mouse Models of Cone-Rod Dystrophy. Investig. Ophthalmol. Vis. Sci..

[71] Singh M., Aslam S., Duncan I., Cramer A., Barnard A., MacLaren R. (2014). Cell fusion following photoreceptor transplantation into the non-degenerate retina. Investig. Ophthalmol. Vis. Sci..

[72] Ortin-Martinez A., Tsai E.L., Nickerson P.E., Bergeret M., Lu Y., Smiley S., Comanita L., Wallace V.A. (2017). A Reinterpretation of Cell Transplantation: GFP Transfer From Donor to Host Photoreceptors. Stem Cells.

[73] Siqueira R.C., Messias A., Messias K., Arcieri R.S., Ruiz M.A., Souza N.F., Martins L.C., Jorge R. (2015). Quality of life in patients with retinitis pigmentosa submitted to intravitreal use of bone marrow-derived stem cells (Reticell -clinical trial). Stem Cell Res. Ther..

[74] Terrell D., Comander J. (2019). Current Stem-Cell Approaches for the treatment of inherited retinal degenerations. Semin. Ophthalmol..

[75] Zheng A., Li Y., Tsang S.H. (2015). Personalized therapeutic strategies for patients with retinitis pigmentosa. Expert Opin. Biol. Ther..

[76] Cai B., Sun S., Li Z., Zhang X., Ke Y., Yang J., Li X. (2018). Application of CRISPR/Cas9 technologies combined with iPSCs in the study and treatment of retinal degenerative diseases. Hum. Genet..

[77] Burnight E.R., Wiley L.A., Mullins R.F., Stone E.M., Tucker B.A. (2014). Gene therapy using stem cells. Cold Spring Harb. Perspect. Med..

[78] Chuang K., Fields M.A., Del Priore L.V. (2017). Potential of Gene Editing and Induced Pluripotent Stem Cells (iPSCs) in Treatment of Retinal Diseases. Yale J. Biol. Med..

[79] Liao H.K., Hatanaka F., Araoka T., Reddy P., Wu M.Z., Sui Y., Yamauchi T., Sakurai M., O’Keefe D.D., Núñez-Delicado E. (2017). In Vivo Target Gene Activation via CRISPR/Cas9-Mediated Trans-epigenetic Modulation. Cell.

[80] Santiago C.P., Keuthan C.J., Boye S.L., Boye S.E., Imam A.A., Ash J.D. (2018). A Drug-Tunable Gene Therapy for Broad-Spectrum Protection against Retinal Degeneration. Mol. Ther..

[81] Cereso N., Pequignot M.O., Robert L., Becker F., De Luca V., Nabholz N., Rigau V., De Vos J., Hamel C.P., Kalatzis V. (2014). Proof of concept for AAV2/5-mediated gene therapy in iPSC-derived retinal pigment epithelium of a choroideremia patient. Mol. Ther. Methods Clin. Dev..

[82] Burnight E.R., Wiley L.A., Drack A.V., Braun T.A., Anfinson K.R., Kaalberg E.E., Halder J.A., Affatigato L.M., Mullins R.F., Stone E.M. (2014). CEP290 gene transfer rescues Leber congenital amaurosis cellular phenotype. Gene Ther..

[83] Bassuk A.G., Zheng A., Li Y., Tsang S.H., Mahajan V.B. (2016). Precision Medicine: Genetic Repair of Retinitis Pigmentosa in Patient-Derived Stem Cells. Sci. Rep..

[84] Garita-Hernandez M., Lampič M., Chaffiol A., Guibbal L., Routet F., Santos-Ferreira T., Gasparini S., Borsch O., Gagliardi G., Reichman S. (2019). Restoration of visual function by transplantation of optogenetically engineered photoreceptors. Nat. Commun..

[85] Drori T., Chapman J. (2014). Diagnosis and classification of neuromyelitis optica (Devic’s syndrome). Autoimmun Rev..

[86] Mallick J., Devi L., Malik P.K., Mallick J. (2016). Update on Normal Tension Glaucoma. J. Ophthalmic Vis. Res..

[87] Kauper K., McGovern C., Sherman S., Heatherton P., Rapoza R., Stabila P., Dean B., Lee A., Borges S., Bouchard B. (2012). Two-year intraocular delivery of ciliary neurotrophic factor by encapsulated cell technology implants in patients with chronic retinal degenerative diseases. Investig. Ophthalmol. Vis. Sci..

[88] Birch D.G., Weleber R.G., Duncan J.L., Jaffe G.J., Tao W. (2013). Ciliary Neurotrophic Factor Retinitis Pigmentosa Study Groups. Randomized trial of ciliary neurotrophic factor delivered by encapsulated cell intraocular implants for retinitis pigmentosa. Am. J. Ophthalmol..

[89] Levison S.W., Ducceschi M.H., Young G.M., Wood T.L. (1996). Acute exposure to CNTF in vivo induces multiple components of reactive gliosis. Exp. Neurol..

[90] Amore G., Romagnoli M., Carbonelli M., Barboni P., Carelli V., La Morgia C. (2021). Therapeutic Options in Hereditary Optic Neuropathies. Drugs.

[91] Zhao T., Li Y., Tang L., Li Y., Fan F., Jiang B. (2011). Protective effects of human umbilical cord blood stem cell intravitreal transplantation against optic nerve injury in rats. Graefes Arch. Clin. Exp. Ophthalmol..

[92] Lopez Sanchez M.I., Crowston J.G., Mackey D.A., Trounce I.A. (2016). Emerging Mitochondrial Therapeutic Targets in Optic Neuropathies. Pharmacol. Ther..

[93] Usategui-Martín R., Puertas-Neyra K., García-Gutiérrez M.T., Fuentes M., Pastor J.C., Fernandez-Bueno I. (2020). Human Mesenchymal Stem Cell Secretome Exhibits a Neuroprotective Effect over *In Vitro* Retinal Photoreceptor Degeneration. Mol. Ther. Methods Clin. Dev..

[94] Labrador-Velandia S., Alonso-Alonso M.L., Di Lauro S., García-Gutierrez M.T., Srivastava G.K., Pastor J.C., Fernandez-Bueno I. (2019). Mesenchymal stem cells provide paracrine neuroprotective resources that delay degeneration of co-cultured organotypic neuroretinal cultures. Exp. Eye Res..

[95] Weiss J.N., Levy S. (2019). Stem Cell Ophthalmology Treatment Study (SCOTS): Bone marrow derived stem cells in the treatment of Dominant Optic Atrophy. Stem Cell Investig..

[96] Nascimento-Dos-Santos G., Teixeira-Pinheiro L.C., da Silva-Júnior A.J., Carvalho L.R.P., Mesentier-Louro L.A., Hauswirth W.W., Mendez-Otero R., Santiago M.F., Petrs-Silva H. (2020). Effects of a combinatorial treatment with gene and cell therapy on retinal ganglion cell survival and axonal outgrowth after optic nerve injury. Gene Ther..

[97] Huang H., Kolibabka M., Eshwaran R., Chatterjee A., Schlotterer A., Willer H., Bieback K., Hammes H.P., Feng Y. (2019). Intravitreal injection of mesenchymal stem cells evokes retinal vascular damage in rats. FASEB J..

[98] Labrador Velandia S., Di Lauro S., Alonso-Alonso M.L., Tabera Bartolome S., Srivastava G.K., Pastor J.C., Fernandez-Bueno I. (2018). Biocompatibility of intravitreal injection of human mesenchymal stem cells in immunocompetent rabbits. Graefes Arch. Clin. Exp. Ophthalmol..

[99] Park S.S., Bauer G., Abedi M., Pontow S., Panorgias A., Jonnal R., Zawadzki R.J., Werner J.S., Nolta J. (2014). Intravitreal autologous bone marrow CD34+ cell therapy for ischemic and degenerative retinal disorders: Preliminary phase 1 clinical trial findings. Investig. Ophthalmol Vis. Sci..

[100] Gu X., Yu X., Zhao C., Duan P., Zhao T., Liu Y., Li S., Yang Z., Li Y., Qian C. (2018). Efficacy and Safety of Autologous Bone Marrow Mesenchymal Stem Cell Transplantation in Patients with Diabetic Retinopathy. Cell Physiol. Biochem..

[101] China. Shanghai General Hospital, Shanghai Jiao Tong University School of Medicine Role of the Serum Exosomal miRNA in Diabetic Retinopathy (DR). https://clinicaltrials.gov/ct2/show/NCT03264976.

[102] Castanheira P., Torquetti L., Nehemy M.B., Goes A.M. (2008). Retinal incorporation and differentiation of mesenchymal stem cells intravitreally injected in the injured retina of rats. Arq. Bras. Oftalmol..

[103] van Zeeburg E.J., Maaijwee K.J., Missotten T.O., Heimann H., van Meurs J.C. (2012). A free retinal pigment epithelium-choroid graft in patients with exudative age-related macular degeneration: Results up to 7 years. Am. J. Ophthalmol..

[104] Ma Z., Han L., Wang C., Dou H., Hu Y., Feng X., Xu Y., Wang Z., Yin Z., Liu Y. (2009). Autologous transplantation of retinal pigment epithelium-Bruch’s membrane complex for hemorrhagic age-related macular degeneration. Investig. Ophthalmol Vis. Sci..

[105] Jung Y.H., Phillips M.J., Lee J., Xie R., Ludwig A.L., Chen G., Zheng Q., Kim T.J., Zhang H., Barney P. (2018). 3D Microstructured Scaffolds to Support Photoreceptor Polarization and Maturation. Adv. Mater..

[106] Boyd A.S., Higashi Y., Wood K.J. (2005). Transplanting stem cells: Potential targets for immune attack. Modulating the immune response against embryonic stem cell transplantation. Adv. Drug Deliv. Rev..

[107] Drukker M., Katz G., Urbach A., Schuldiner M., Markel G., Itskovitz-Eldor J., Reubinoff B., Mandelboim O., Benvenisty N. (2002). Characterization of the expression of MHC proteins in human embryonic stem cells. Proc. Natl. Acad. Sci. USA.

[108] Araki R., Uda M., Hoki Y., Sunayama M., Nakamura M., Ando S., Sugiura M., Ideno H., Shimada A., Nifuji A. (2013). Negligible immunogenicity of terminally differentiated cells derived from induced pluripotent or embryonic stem cells. Nature.

[109] Sugita S., Iwasaki Y., Makabe K., Kamao H., Mandai M., Shiina T., Ogasawara K., Hirami Y., Kurimoto Y., Takahashi M. (2016). Successful Transplantation of Retinal Pigment Epithelial Cells from MHC Homozygote iPSCs in MHC-Matched Models. Stem Cell Rep..

[110] Umekage M., Sato Y., Takasu N. (2019). Overview: An iPS cell stock at CiRA. Inflamm. Regen..

[111] Sugita S., Mandai M., Hirami Y., Takagi S., Maeda T., Fujihara M., Matsuzaki M., Yamamoto M., Iseki K., Hayashi N. (2020). HLA-Matched Allogeneic iPS Cells-Derived RPE Transplantation for Macular Degeneration. J. Clin. Med..

[112] Hazra S., Stepps V., Bhatwadekar A.D., Caballero S., Boulton M.E., Higgins P.J., Nikonova E.V., Pepine C.J., Thut C., Finney E.M. (2013). Enhancing the function of CD34(+) cells by targeting plasminogen activator inhibitor-1. PLoS ONE.

[113] Ueki Y., Wilken M.S., Cox K.E., Chipman L., Jorstad N., Sternhagen K., Simic M., Ullom K., Nakafuku M., Reh T.A. (2015). Transgenic expression of the proneural transcription factor Ascl1 in Müller glia stimulates retinal regeneration in young mice. Proc. Natl. Acad. Sci. USA.

[114] Mesentier-Louro L.A., Teixeira-Pinheiro L.C., Gubert F., Vasques J.F., Silva-Junior A.J., Chimeli-Ormonde L., Nascimento-Dos-Santo G., Mendez-Otero R., Santiago M.F. (2019). Long-term neuronal survival, regeneration, and transient target reconnection after optic nerve crush and mesenchymal stem cell transplantation. Stem Cell Res. Ther..

[115] Barber A., Farmer K., Martin K.R., Smith P.D. (2017). Retinal regeneration mechanisms linked to multiple cancer molecules: A therapeutic conundrum. Prog. Retin. Eye Res..

[116] Mead B., Tomarev S. (2017). Bone Marrow-Derived Mesenchymal Stem Cells-Derived Exosomes Promote Survival of Retinal Ganglion Cells Through miRNA-Dependent Mechanisms. Stem Cells Transl. Med..

[117] Gramlich O.W., Brown A.J., Godwin C.R., Chimenti M.S., Boland L.K., Ankrum J.A., Kardon R.H. (2020). Systemic Mesenchymal Stem Cell Treatment Mitigates Structural and Functional Retinal Ganglion Cell Degeneration in a Mouse Model of Multiple Sclerosis. Transl. Vis. Sci. Technol..

